# Importance of applying Mixed Generalized Additive Model (MGAM) as a method for assessing the environmental health impacts: Ambient temperature and Acute Myocardial Infarction (AMI), among elderly in Shanghai, China

**DOI:** 10.1371/journal.pone.0255767

**Published:** 2021-08-12

**Authors:** Xiaoqian Huang, Weiping Ma, Chikin Law, Jianfeng Luo, Naiqing Zhao

**Affiliations:** 1 Department of Biostatistics, School of Public Health, Fudan University, Shanghai, China; 2 NHC Key Laboratory of Health Technology Assessment, Fudan University, Shanghai, China; 3 Key Laboratory of Public Health Safety of Ministry of Education, Fudan University, Shanghai, China; 4 Department of Genetics and Genomic Sciences, Icahn School of Medicine at Mount Sinai, New York, NY, United States of America; 5 Icahn Institute of Genomics and Multiscale Biology, Icahn School of Medicine at Mount Sinai, New York, NY, United States of America; 6 NHMRC Clinical Trials Centre, Faculty of Medicine and Health, The University of Sydney, Sydney, Australia; Universidad Miguel Hernandez de Elche, SPAIN

## Abstract

Association between acute myocardial infarction (AMI) morbidity and ambient temperature has been examined with generalized linear model (GLM) or generalized additive model (GAM). However, the effect size by these two methods might be biased due to the autocorrelation of time series data and arbitrary selection of degree of freedom of natural cubic splines. The present study analyzed how the climatic factors affected AMI morbidity for older adults in Shanghai with Mixed generalized additive model (MGAM) that addressed these shortcomings mentioned. Autoregressive random effect was used to model the relationship between AMI and temperature, PM_10_, week days and time. The degree of freedom of time was chosen based on the seasonal pattern of temperature. The performance of MGAM was compared with GAM on autocorrelation function (ACF), partial autocorrelation function (PACF) and goodness of fit. One-year predictions of AMI counts in 2011 were conducted using MGAM with the moving average. Between 2007 and 2011, MGAM adjusted the autocorrelation of AMI time series and captured the seasonal pattern after choosing the degree of freedom of time at 5. Using MGAM, results were well fitted with data in terms of both internal (R^2^ = 0.86) and external validity (correlation coefficient = 0.85). The risk of AMI was relatively high in low temperature (Risk ratio = 0.988 (95% CI 0.984, 0.993) for under 12°C) and decreased as temperature increased and speeded up within the temperature zone from 12°C to 26°C (Risk ratio = 0.975 (95% CI 0.971, 0.979), but it become increasing again when it is 26°C although not significantly (Risk ratio = 0.999 (95% CI 0.986, 1.012). MGAM is more appropriate than GAM in the scenario of response variable with autocorrelation and predictors with seasonal variation. The risk of AMI was comparatively higher when temperature was lower than 12°C in Shanghai as a typical representative location of subtropical climate.

## Introduction

Generalized linear model (GLM) and generalized additive model (GAM) are the two most commonly used statistical methods to analyze the relationship between environmental factors with epidemiological outcomes [1–4]. However, both GLM and GAM with existed model fitting framework might not appropriately fit time series data in environmental epidemiological studies.

Acute myocardial infarction (AMI) is a life-threatening condition, which affects more than 7 million individuals worldwide annually and causes over one-third of deaths in developed countries [5–7]. The global burden of cardiovascular diseases, including myocardial infarction will be rocketed up in developing countries due to huge population size and aging society [8–10].

Among the spectrum of risk factors of AMI, ambient temperature has attracted many interest of society [11, 12] in the era of climate change. However, the association between ambient temperature and AMI remains unclear and inconsistent [13–15]. The inconsistence may be attributed to various sources of data, inconsistent AMI ascertainment, and use of different statistical methodologies [11, 12].

As the response variable in a time-dependent model, AMI count is a time series data characterized with auto-correlated patterns, which does not follow the independence assumption for GLM and GAM. Also, when accounting the degree of freedom (df) in the natural spine using GAM, df is often arbitrarily set at 4 or 7 per year in previous studies [3, 16, 17]. As reported in our previous studies, this arbitrary rule would heighten the risk of over fitting [18, 19]. Due to the weak association between ambient temperature and AMI [20, 21], the estimation bias caused by arbitrary rule of GAM and GLM would be considered improper that cannot be ignored.

Mixed generalized additive model (MGAM), with an autoregressive term in random effect, offers a better alternative for data analysis in environmental epidemiological study. Besides of it, we have developed a robust strategy to determine the degrees of freedom of natural splines in MGAM [19, 22–24].

Additionally, most of recent studies on the association between temperature and risk of AMI were conducted in high latitude areas since the cold weather triggers cardiovascular diseases of different types [25]. Very few studies have been conducted in metropolitan areas with sub-tropical climate like the Municipality of Shanghai [15].To manifest the performance of MGAM in the scenario of time series environmental epidemiological data, MGAM and GAM will be compared on the modeling of ambient temperature and AMI morbidity in Shanghai, China.

## Methods

### Ethical statement

As aggregated data with no personal information were involved, ethical review was exempted by the Institutional Review Board of the Public Health School, Fudan University.

### Data

Shanghai is situated in the central-eastern China with north subtropical monsoon climate. It is a density metropolis with a total resident population of 19.2 million according to the 2010 national census [26].

The daily number of emergency department (ED) attendances by AMI from 2007 to 2011 were obtained from the official Medicare Database in Shanghai. All the researcher can only access to aggregated daily number of AMI by sex and age rather than any identifying AMI patient information. Under the Tenth Revision of the International Classification of Diseases (ICD-10), AMI was defined as I21 and I22.913. Since the population size in Shanghai during 2007 to 2011 was stable, daily AMI cases, rather than incidence rate of AMI, was used as the response variable in GAM or MGAM in the present study. Only AMI patients aged 65 years or above were included since we thought those subjects were vulnerable to the impact of ambient temperature. The official Medicare Database records all ED attendances among members of Shanghai’s social health insurance, which included usual residents with Shanghai’s household registration or persons with paid employment contract of more than six months.

Meteorological index, including daily averaged ambient temperature and relative humidity, were retrieved from the Shanghai Meteorological Bureau. Daily concentrations of particulate matter 10 micrometers or less in diameter (PM_10_), sulphur dioxide (SO_2_) and nitrogen dioxide (NO_2_) were obtained from the Shanghai Environmental Monitoring Center.

The population sizes of Shanghai residents age 65+ from Jan 2007 to Jan 2012 were collected from the Shanghai Research Center on Aging.

### Statistical methods

Mean, standard deviation, minimum and maximum were used to describe the count of AMI, ambient temperature, relative humidity and air pollutants for whole examined period, as well as for all seasons. Pearson correlation were applied for the correlation among ambient temperature, relative humidity and air pollutants. Spearman correlation were applied for the correlation between ambient temperature and count of AMI.

GAM and MGAM [27] were used to analyze the statistical association between ambient temperature, relative humidity, air pollutants, week days and AMI morbidity. The detailed methodology was described elsewhere [22, 23] and also in the supplement.

The most important step was using GAM to estimate the degree of freedom of the natural spline function for time, NS(t, df_t_). The seasonal pattern of temperature effect was used to determine suitable degree of freedom to control those unmeasured factors in (NS(t, df_t_)) and thus achieve the unbiased estimation of temperature effect. After the selection of df of time, the Akaike information criterion was applied to determine the degree of freedom of other factors such as df_temp_ and df_pm10_. The coefficients and weights for temperature were estimated by maximum partial likelihood using Newton’s Method. Variable selection was based on the statistical significance (p value) and professional rationale. The model with ambient temperature and PM_10_ as independent variables was selected based on the Akaike information criterion using GAM. After the modeling of spline, risk ratio and confidence interval was approximately calculated for 3 temperature zones based on the change point of spline.

The four seasons were classified as spring (March to May), summer (June to August), autumn (September to November) and winter (December to February) as usual [19].

All statistical analyses were conducted using R software (version 3.6.2) and p<0.05 was considered as statistically significant.

## Results

### Descriptive statistics

In Shanghai, over 161,000 elderly men (aged 65+) and 195,000 elderly women attended hospital’s ED by AMI from 2007-01-01 to 2011-12-31 in the official Medicare Database. For elderly men, the annual number of AMI increased from 27.6 thousands in 2007 to 35.6 thousands in 2011, with the mortality rate increased from 29.0 to 33.0 per 1,000 population. For elderly women, the annual number of AMI increased from 34.3 thousands in 2007 to 42.1 thousands in 2011, with the rate increased from 29.5 to 33.1 per 1000. (Table 1).

**Table 1:** Numbers and rates of acute myocardial infarction in Shanghai residents aged 65+ by gender, 2007–2011.

	Males			Females		
Year	Population	Number of AMI	Rates	Population	Number of AMI	Rates
	(thousands)	(thousands)	(per1,000)	(thousands)	(thousands)	(per1,000)
2007	950	27.6	29.0	1160	34.3	29.5
2008	971	31.2	32.1	1170	38.1	32.4
2009	1006	31.7	31.5	1200	38.2	31.7
2010	1034	35.3	34.1	1230	42.9	34.9
2011	1080	35.6	33.0	1270	42.1	33.1

**Fig 1:**
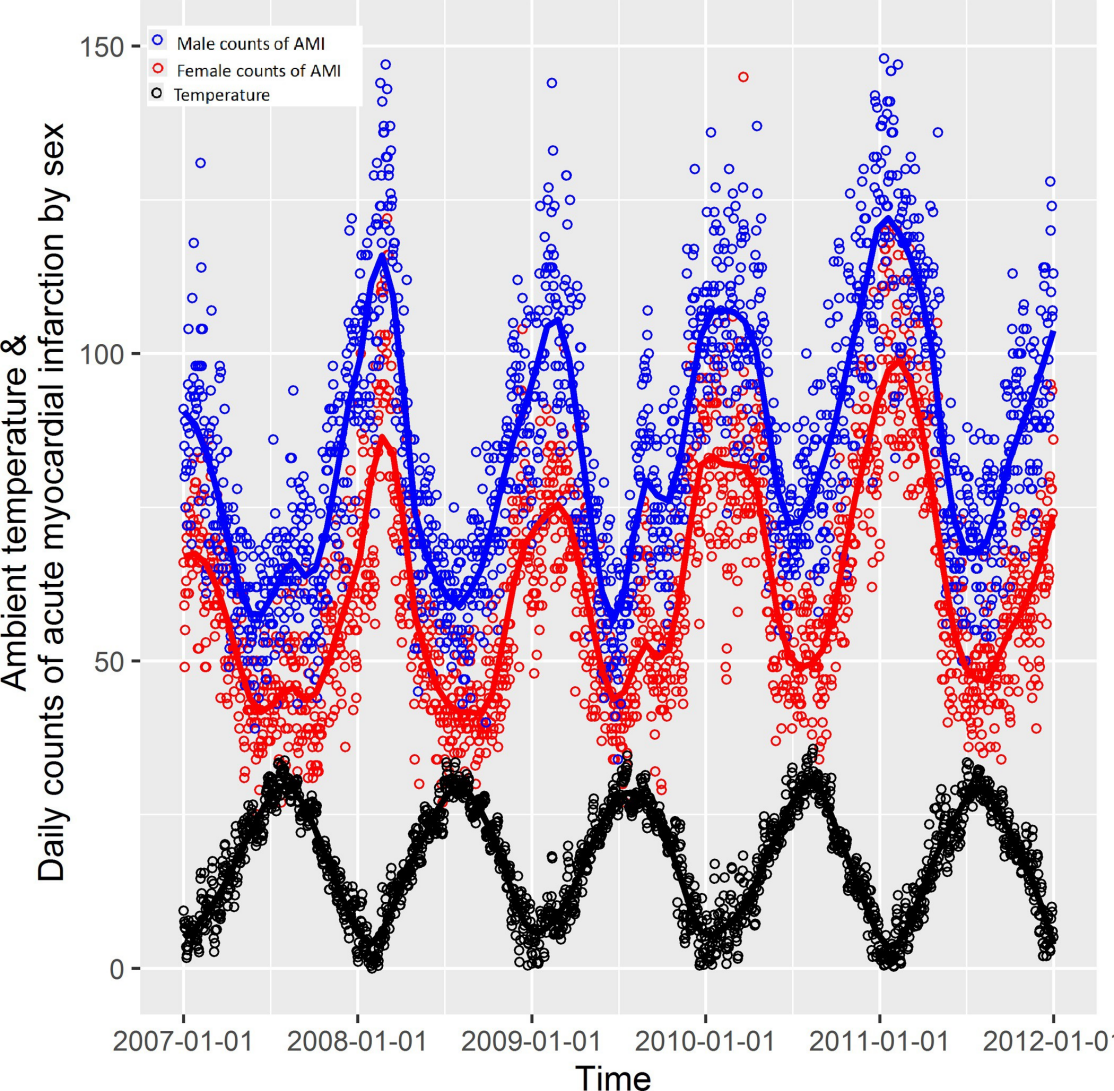
Time series of daily cases of Acute Myocardial Infarction (AMI) by sex and ambient temperature in Shanghai from 2007 to 2011.

The ambient temperature within the study period was with daily mean temperature around 17.3°C and 17.0°C in spring, 27.7°C in summer, 19.8°C in autumn and 6.0°C in winter. The mean of PM_10_ within the study period was around 80.5 μg/m^3^ and 88.6 μg/m^3^ in spring, 64.6 μg/m^3^ in summer, 75.3 μg/m^3^ in autumn and 90.5 μg/m^3^ in winter (Table 2). The description of relative humidity, SO_2_, NO_2_ was also listed in Table 2. The ambient temperature, relative humidity and air pollutants were correlated with statistical significance (Table 3). Daily numbers of AMI had negative correlation with temperature (Spearman correlation = −0.4, p<0.05, Fig 1).

**Table 2:** The description of ambient temperature, relative humidity, air pollutants during 2007–2011*.

	Season	mean	standard deviation	minimum	maximum
Temperature (°C)	spring	17.0	6.5	1.60	32.80
	summer	27.7	3.2	19.80	35.70
	autumn	19.8	5.5	4.10	31.00
	winter	6.0	3.9	-3.40	18.20
	whole Year	17.3	9.1	-3.40	35.70
relative humidity (%)	spring	66.2	14.4	30.00	94.00
	summer	74.3	8.6	49.00	95.00
	autumn	70.2	10.6	35.00	93.00
	winter	68.3	13.2	23.00	95.00
	whole Year	69.4	12.5	23.00	95.00
SO_2_ (μg /m^3^)	spring	34.7	21.5	6	114
	summer	29.0	16.5	7	97
	autumn	34.1	20.4	9	119
	winter	53.7	33.7	11	229
	whole Year	37.8	25.5	6	229
NO_2_ (μg/m^3^)	spring	52.9	19.4	11	116
	summer	40.6	16.6	11	122
	autumn	52.3	21.9	16	132
	winter	61.1	21.0	22	155
	whole Year	51.9	21.0	11	155
PM_10_ (μg /m^3^)	spring	88.6	68.4	17	792
	summer	64.6	34.3	10	251
	autumn	75.3	50.3	16	476
	winter	90.5	58.2	14	513
	whole Year	80.5	56.4	10	792

* PM_10_: particulate matter 10 micrometers or less in diameter, SO_2_: sulphur dioxide and NO_2_: nitrogen dioxide.

**Table 3:** Pearson correlation among ambient temperature, relative humidity and air pollutants*.

	temperature	relative humidity	SO_2_	NO_2_	PM_10_
Temperature	1	.180**	-.354**	-.372**	-.157**
relative humidity		1	-.350**	-.206**	-.310**
SO_2_			1	.694**	.575**
NO_2_				1	.620**
PM_10_					1

* PM_10_: particulate matter 10 micrometers or less in diameter, SO_2_: sulphur dioxide and NO_2_: nitrogen dioxide.

** p<0.001.

### Model fitting

**Fig 2:**
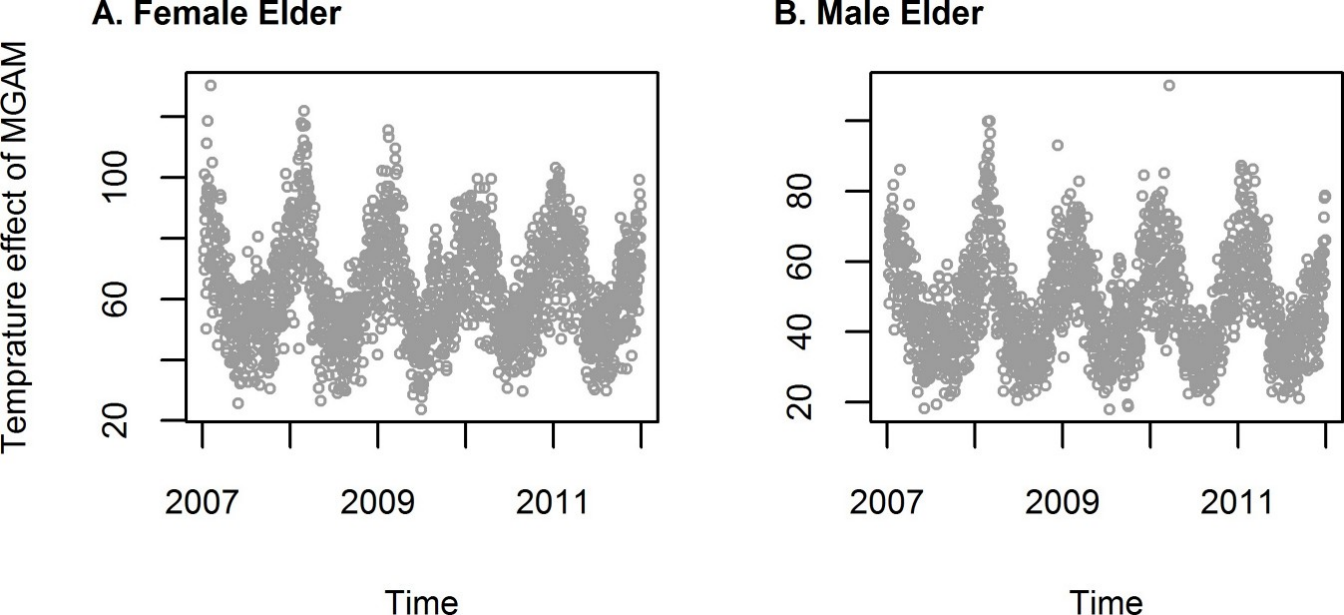
Seasonal pattern of temperature after proper selection of degree of freedom of natural spline (df_t_ = 5).

For both female and male AMI counts, df_t_ = 5 and df_temp_ = 5 were determined using the methods described in the previous section. The degree of freedom of natural spline was selected because the MGAM model we adopted shown seasonal pattern of temperature for both female and male (Fig 2).

**Fig 3:**
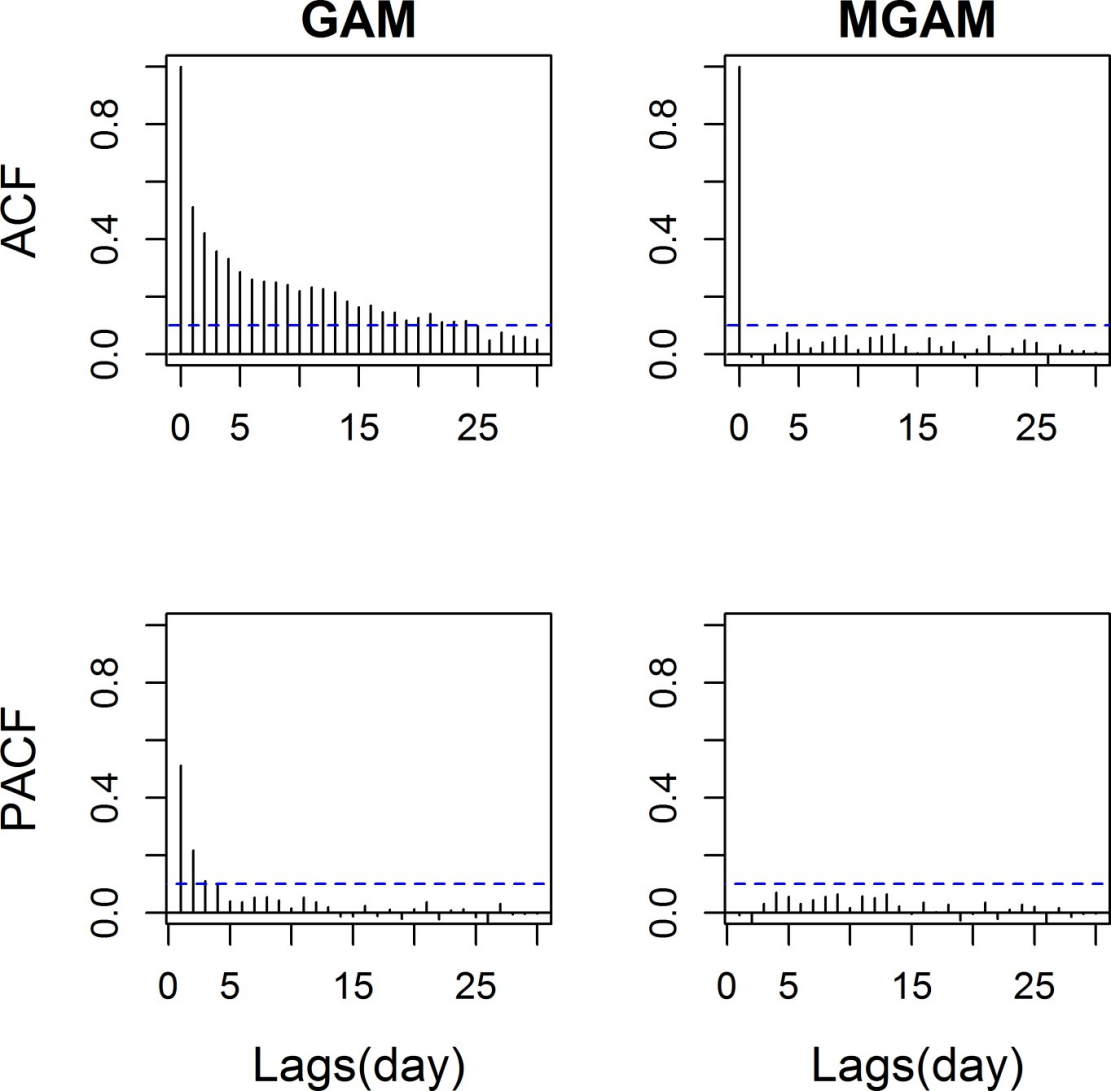
Autocorrelation function (ACF) and partial autocorrelation function (PACF) of GAM and MGAM model (df_t_ = 5 and df_temp_ = 5).

From the residual plots of ACF and PACF, the autocorrelation and partial autocorrelation coefficients of GAM exceeded the uncorrelated criteria 0.10 for some nonzero lags (Fig 3). However, the autocorrelation and partial autocorrelation coefficients of MGAM did not exceed 0.10 for all nonzero lags (Fig 3) with the autocorrelation order p = 2.

**Fig 4:**
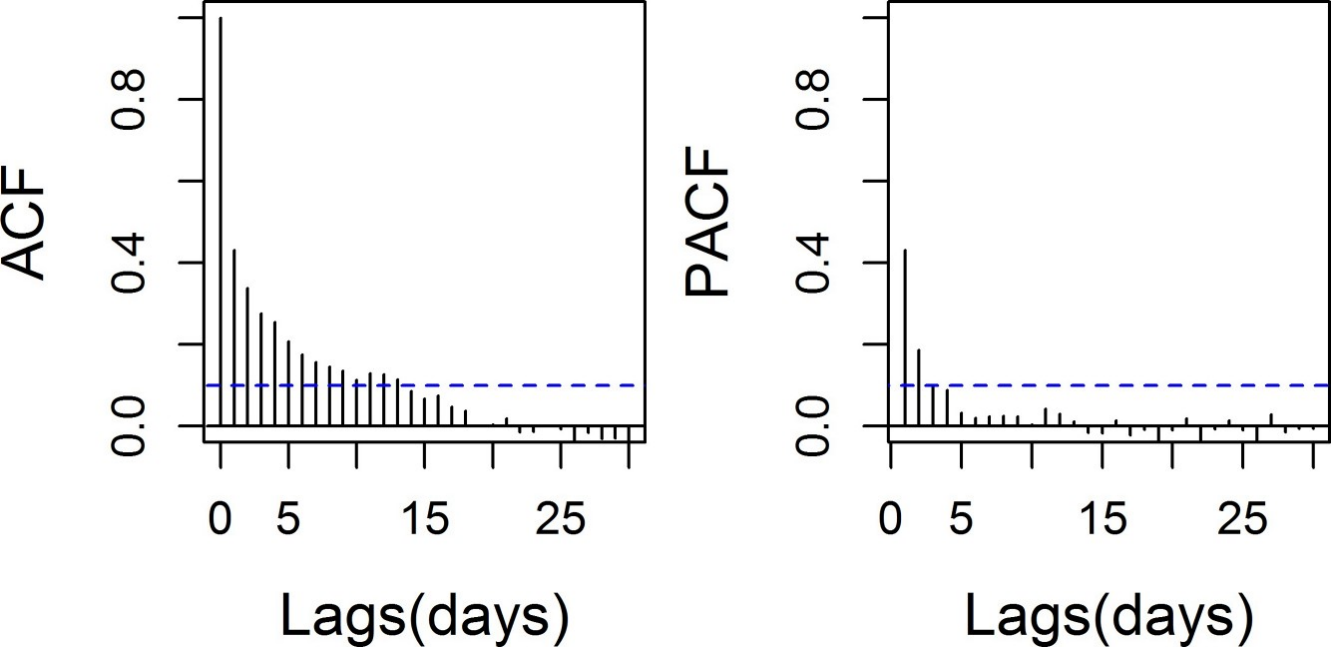
Autocorrelation function (ACF) and partial autocorrelation function (PACF) of GAM (df_t_ = 20 and df_temp_ = 5).

**Fig 5:**
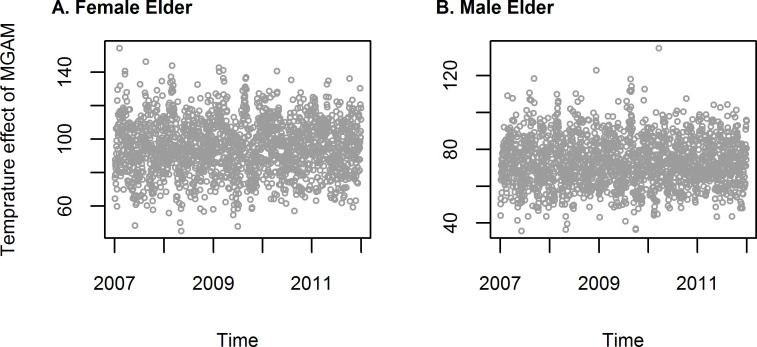
No seasonal pattern temperature after arbitrary set of degree of freedom of natural spline at 4 per year (df_t_ = 20).

If we arbitrarily set the degree of freedom of natural spline at 4 per year, this equals to 20 for a 5-year period from 2007 to 2011. With the increasing of df, GAM handled the autocorrelation issue better than df = 5 (Fig 4 compared with Fig 3). But no seasonal pattern temperature would be found which indicated over-fitting (Fig 5).

**Fig 6:**
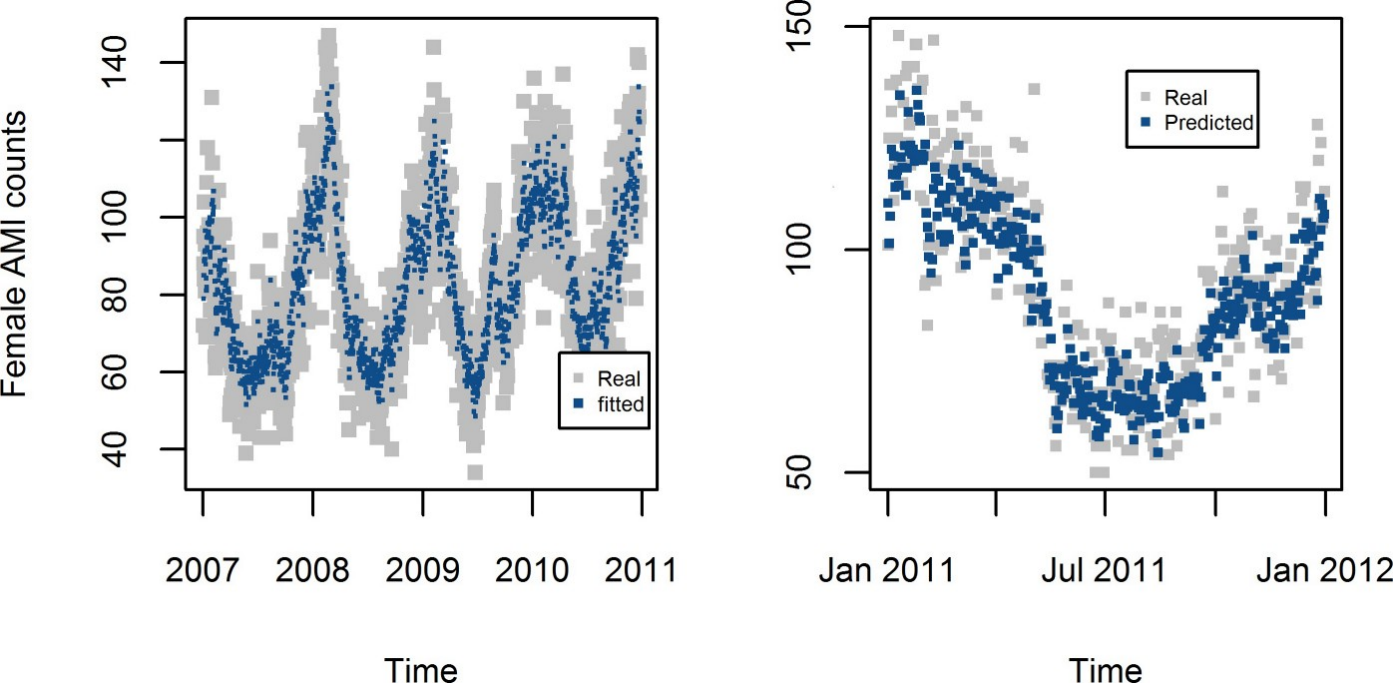
Estimated and forecasted daily counts of AMI from MGAM in Shanghai female residents aged 65+.

**Fig 7:**
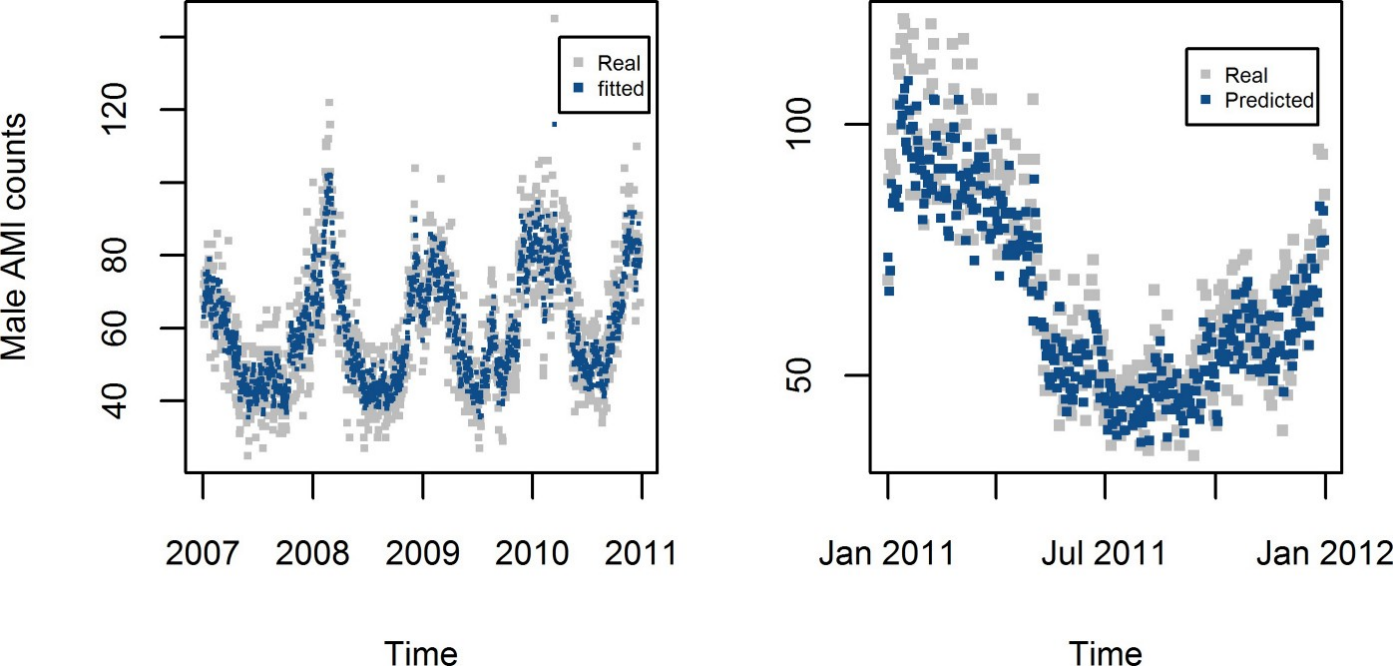
Estimated and forecasted daily counts of AMI from MGAM in Shanghai male residents aged 65+.

The degree of freedom in the spline function was 5 for both time and temperature, and the order of the autocorrelation was 2. The models derived by MGAM were well fitted for the female AMI data (R^2^ = 0.860) and male AMI data (R^2^ = 0.856) (Figs 6 and 7).

### Association between ambient temperature and AMI counts

**Fig 8:**
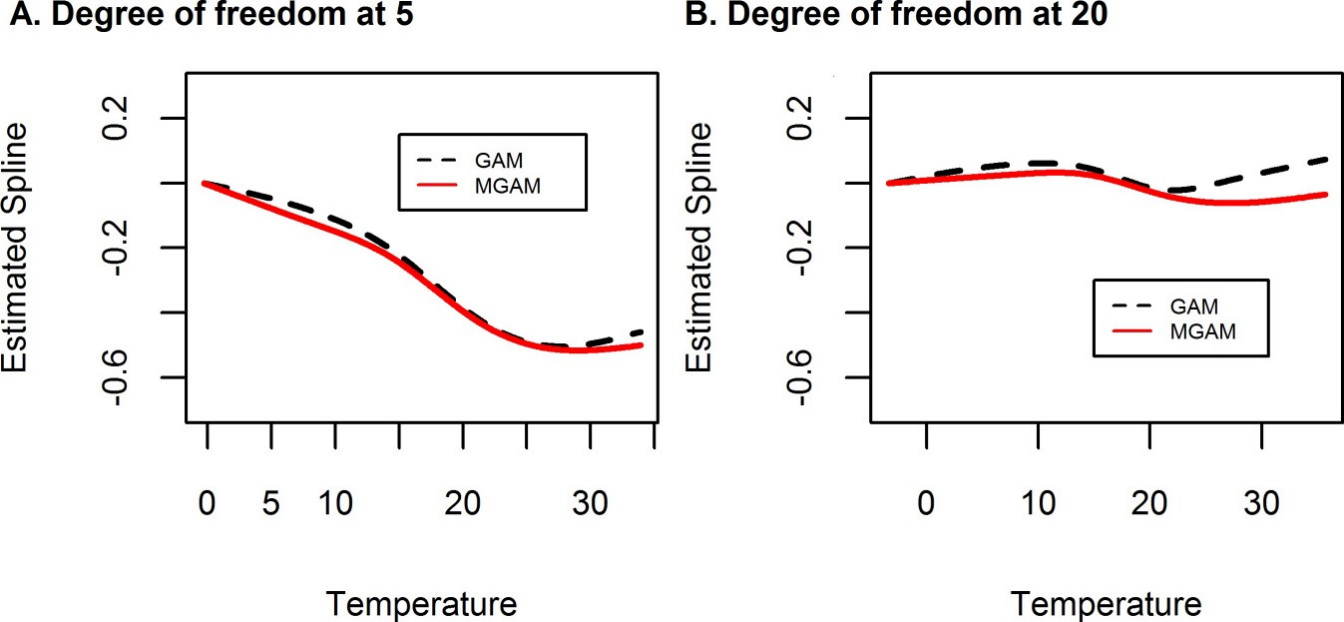
Estimated spline for temperature effect on AMI in Shanghai female residents aged 65+, 2007–2011.

Since both genders shared similar patterns, only the association between ambient temperature and AMI counts of female was presented here. The relationship between the risk of AMI with ambient temperature was manifested as a mirror image of J-shape curve within the range of temperature in this study. The risk of AMI was relatively high in low temperature (Risk ratio = 0.988 (95% CI 0.984, 0.993) for under 12°C) and decreased as temperature increased and speeded up within the temperature zone from 12°C to 26°C (Risk ratio = 0.975 (95% CI 0.971, 0.979), but it become increasing again when it is 26°C although not significantly (Risk ratio = 0.999 (95% CI 0.986, 1.012) (Fig 8A). For GAM model with df set at 4 per year, equal to df = 20 for 5 years, no association could be found (Fig 8B).

### Forecast the risk of AMI in 2011

Predicted AMI counts in 2011with the model established using data from 2007 to 2010 has good consistence with the original AMI counts in the scatter plots in Fig 8 with Spearman correlation coefficient 0.86 and 0.85 for female and male respectively (Figs 6 and 7).

## Discussion

MGAM used in this present study successfully incorporated the autocorrelation effect of daily AMI cases in consecutive days. Concurrently, we proposed a robust strategy to select the degree of freedom on the time series predictor, which is controversial in other widely used GLM or GAM [1–4]. The time effect (NS(t, df_t_)) in our model was used to control those unmeasured nuisances. With the increase of df_t_, the time effect alone can fit exactly well with response variable and erase the effect of all the other factors. To maintain the seasonal pattern of temperature factor in the model, we can pick the properly lowest degree of freedom of time. On the other hand, specific lag effect chosen subjectively or by time series methods for forecasting [12–14] have risk on over-fitting (Fig 5) which could erase the association between exposure and disease (Fig 8) In the present study, weighted average of daily mean temperature in the past 7 days was used to model the lag effect of ambient temperature with AMI. Our model was more appropriate as it does not only take into account the effect of average temperature, but also the impulse impact of some particular temperature [19, 22, 23].

Our study indicates that in general, lower temperature is a risk factor for the incidence of acute myocardial infarction among elderly in Shanghai. Both older men and older women had mirror image J-shape association between ambient temperature and the incidence of AMI. When temperature stayed below 12°C, incidence of AMI was relatively high, and as the temperature increased above 12°C the risk decreased. However, the trend was reversed when the temperature exceeded 26°C, AMI incidence seems increasing as the temperature rose. To our knowledge, this is the first study on this topic in a warm climate city using an appropriate statistical method, MGAM.

Results of this study were consistent with previous findings from Hong Kong and Taiwan, where temperature below a threshold of 24°C was significantly associated with AMI hospitalization but no significant heat effects were found [15]. Low temperature exposure as a risk factor for AMI were also found in the Worcester metropolitan area, MA, US [14] and in Belgium [28]. A 10-year longitudinal study also found that rates of myocardial infarction events decreased with increasing atmospheric temperature [29]. However, low temperature exposure was not found to be a major triggering factor of myocardial infarctions in cold area like Sweden [30] and Minnesota, US [31]. The U-shape association between temperature and risk of AMI was found in Korea [13] and the Hunter Region of New South Wales in Australia [32]. Inferred from these studies, the association between ambient temperature with AMI might has various pattern in different regions. Residents of different regions might have already adapted to their habitat’s weather, but remain sensitive to stress caused by extreme temperature. In an analysis on 21 countries registry database, rates of coronary events increased during comparatively cold periods, especially in warm area [33]. These findings indicate that both the normal range of temperature and extreme weather of the specific area should be considered in the analysis of the impact of temperature on human health. In this era of climate change and extreme weather, residents and policy maker should prepare for it, especially for vulnerable population.

Several possible mechanisms can explain why low temperature exposure could increase the risk of AMI. Cold stress is known to result in vasoconstriction and blood pressure rises [34, 35] which is one of the most prominent risk factors for myocardial infarction, especially in patients with essential hypertension [36]. Additionally, increased sympathetic nervous activity and an increased load of sodium presented to the kidney for excretion in winter may also increase the risk of AMI [36]. Low temperature exposure may also impact the haemostatic system by increasing platelet counts and its sensitivity in whole blood and hence the plasma viscosity [34, 35].

Humidity, air pollutants like SO_2_, NO_2_, especially PM_10_ were found to be a risk factor for disease like AMI. However, these factors were not significant in our MGAM model. It might be explained by the correlation between these factors and ambient temperature in our study.

Some limitations should be considered for the present study. First is the ecological fallacy. Although the natural cubic spline (NS(t)) controlled the potential effect of some unmeasured factors, but it is impossible to completely rule out the bias in the ecologic study design. Secondly, PM_2.5_ was commonly used as a confounder in previous environmental epidemiology studies. However, this variable was not available in the current study. Third, there was potential disease misclassification for AMI despite the chance is low. Fourth, we can’t do stratified analysis by subtypes of ST elevation MI (STEMI) and NSTEMI. Fifth, we use the AMI cases reached for the emergency visits reported in the Official Medicare Database. It will miss some mild AMI or some severe AMI cause sudden death.

## Conclusion

MGAM is more appropriate than GAM for time series studies of environmental impact on health, such as effects of ambient temperature on acute myocardial infarction morbidity. Low temperature less than 12°C is a risk factor of AMI morbidity in north subtropical monsoon climate. Our finding enhanced the knowledge about association between the change in temperature and incidence of AMI.

## Supporting information

S1 File The definition of MGAM and prediction model. (DOCX)

S1 Data Meteorological. (CSV)

S2 Data MI. (CSV)
