# Supplementary material for: Importance of applying Mixed Generalized Additive Model (MGAM) as a method for assessing the environmental health impacts: Ambient temperature and Acute Myocardial Infarction (AMI), among elderly in Shanghai, China
Source: PLoS One. 2021 Aug 12;16(8):e0255767. doi: 10.1371/journal.pone.0255767 (PMC8360529; doi:10.1371/journal.pone.0255767)
Supplement: S1 File — (DOCX) [file pone.0255767.s001.docx]

MGAM was defined as follows in the model estimation stage:

$\left\{\begin{matrix} y_{t}\sim Poisson(\mu_{t}) \\ \ln(\mu_{t})=f_{t}+\tau_{t} \\ f_{t}=NS(t,df_{t})+NS(temp_{t},df_{temp})+NS(PM10_{t},df_{pm10})+DOW_{t} \\ \tau_{t}=\sum_{i=1}^{p} \phi_{i}(\ln(y_{t-i}^{*})-f_{t-i}) \\ y_{t-i}^{*}=\max(y_{t-i},0.5) \end{matrix}\right.$ (1)

y_t_ was the number of daily AMI cases assumed to follow Poisson distribution with mean μ_t_.

NS(t, df_t_) was the natural cubic spline of time which accounted for unmeasured time-variant factors such as demographic change, living status and other confounding factors

temp_t_ was the weighted average of daily mean temperature considering the lag effects of temperature in the past week(t-1 to t-7), represented as$temp_{t}=\sum_{i=1}^{7} w_{i}\times temperature_{t-i}$and $\sum_{i=1}^{7} w_{i}=1$. Weighted average temperature was included into the model using natural cubic spline to account for association between temperature with AMI morbidity.

NS(pm10, df_pm10_) was the natural cubic spline of PM10.

DOW_t_ was a dummy variable for day of the week

τ_t_ was the random effect to account for the dependence nature of time series data.

φi was the coefficient of autoregressive random effect.

In the stage of prediction, model was defined as:

$\ln(\hat{\mu}_{t})=\hat{f}_{t}+\tau_{t}$ (2)

with fixed terms:

$\begin{matrix} \hat{f}_{t}=\widehat{\alpha}+NS(\bar{y}_{t-1:t-7},df_{\bar{y}})+NS(temp_{t},df_{temp})+\widehat{DOW_{t}} \\ =\widehat{\alpha}+\widehat{\beta}_{1}\times\ln(\frac{1}{7}\sum_{j=1}^{7} y_{t-j})+\widehat{\beta}_{2}\times\ln(\frac{1}{7}\sum_{j=1}^{7} y_{t-j})^{2}+\sum_{k=0}^{3} \widehat{\beta}_{3+k}\times\left[\ln(\frac{1}{7}\sum_{j=1}^{7} y_{t-j})-y_{k}^{knots}\right]^{3} \\ +\widehat{\gamma}_{1}\times tempt+\widehat{\gamma}_{2}\times temp_{t}^{2}+\sum_{k=0}^{5} \widehat{\gamma}_{3+k}\times(tempt-temp_{k}^{knots})^{3}+\widehat{DOW}_{t} \end{matrix}$ (3)

autoregressive terms:

$\tau_{t}=\sum_{i=1}^{p} \phi_{i}(\ln(y_{t-i}^{*})-f_{t-i})$ (4)
